# Deep intelligence: a four-stage deep network for accurate brain tumor segmentation

**DOI:** 10.1038/s41598-025-18879-x

**Published:** 2025-10-07

**Authors:** Nirmala Paramanandham, Kishore Rajendiran, L. K. Pavithra, M. Niranjan, Tom Michael Shibu, Ashwin Santosh, Aakash Kumar

**Affiliations:** 1 Vellore Institute of technology, Chennai Campus, Chennai, India; 2 Sri Sivasubramaniya Nadar College of Engineering, Kalavakkam, India; 3 Visual Computing, Saarland University, Saarbrücken, Germany

**Keywords:** Image segmentation, Gliomas, Deep learning, Context boosting framework, Brain images, Biomedical engineering, Electrical and electronic engineering

## Abstract

Image segmentation is an essential research field in image processing that has developed from traditional processing techniques to modern deep learning methods. In medical image processing, the primary goal of the segmentation process is to segment organs, lesions or tumors. Segmentation of tumors in the brain is a difficult task due to the vast variations in the intensity and size of gliomas. Clinical segmentation typically requires a high-quality image with relevant features and domain experts for the best results. Due to this, automatic segmentation is a necessity in modern society since gliomas are considered highly malignant. Encoder-decoder-based structures, as popular as they are, have some areas where the research is still in progress, like reducing the number of false positives and false negatives. Sometimes these models also struggled to capture the finest boundaries, producing jagged or inaccurate boundaries after segmentation. This research article introduces a novel and efficient method for segmenting out the tumorous region in brain images to overcome the research gap of the recent state-of-the-art deep learning-based segmentation approaches. The proposed 4-staged 2D-VNET + + is an efficient deep learning tumor segmentation network that introduces a context-boosting framework and a custom loss function to accomplish the task. The results show that the proposed model gives a Dice score of 99.287, Jaccard similarity index of 99.642 and a Tversky index of 99.743, all of which outperform the recent state-of-the-art techniques like 2D-VNet, Attention ResUNet with Guided Decoder (ARU-GD), MultiResUNet, 2D UNet, Link Net, TransUNet and 3D-UNet.

## Introduction

According to the recent survey^1^, a brain tumor is one of the most fatal diseases that can strike people of any gender, including adults and children. Brain tumors are the primary cause of the majority (85–90%) of all primary central nervous system malignancies discovered in the brain. In 2020, it was forecast that 3,08,102 new instances of primary brain or spinal cord tumors would be identified globally. From the above quotes, the role of proper diagnosis and segmentation of the tumors, in the healthcare industry, can be understood.

The 10th most prevalent type of cancer among Indians in 2020 was a brain tumor^2^. According to the International Association of Cancer Registries (IARC), over 28,000 cases of brain tumors are recorded in India every year, and over 24,000 people are reported to have died from brain tumors.

Brain tumor incidence is increasing substantially in India. Each year, a rising number of reports of brain tumors among persons of various ages have been identified. Brain tumors are detected in 40,000 to 50,000 persons annually, 20% of whom are children.

Segmentation of brain tumor or lesion and prediction of the patient’s possible recovery is considered crucial for tumor diagnosis and treatment monitoring. There are several research works and competitions being conducted by top medical and research institutes to improve the algorithms and techniques for the segmentation of brain images. But in order to improve the segmentation of tumors, the image quality matters a lot. Deep learning methods, especially at the signal processing phase, are now redefining medical diagnostic systems. Artificial neural networks are increasingly recommended as a way to support human medical decision-making because of their growing dependability and accuracy. In medical image processing, analysing and improving image quality by segmenting the necessary part are some challenging tasks that have been the subject of a lot of research and development. A lot of contributions have been made in multi-modal image fusion^3–6^. They have been proven effective in the segmentation of these fused images, which are used to provide a diagnosis. But there have been very few attempts shown in storing these enhanced images on a periodical basis, which, when reaching a relevant number of images, can then be stored and used to train the model. This will give a more precise and graphic view of the tumor, and will be extremely useful for assisting surgeries and other invasive medical procedures for removing the tumor.

This research work puts forward a model that can perform brain tumor segmentation efficiently and accurately. A context boosting framework (CBF) helps enhance the underlying textures and contextual features of the image, which exposes more precise areas of the image to be classified as tumorous. This is intended to enhance the accuracy of the fine boundaries. The custom loss function Log Cosh Focal Tversky (LCFT) that employs Focal Tversky loss^7^ and Log Cosh Dice loss^8^ makes sure that the CBF does not enhance the noise in the images, while also helping the model learn the hard examples and extreme cases. The introduction of these two novel concepts has helped reduce the complexity of the model significantly. Consolidating, the major contributions of this research are:

- Introducing a novel method for segmenting tumors from brain MRIs.
- Introducing a customised loss function LCFT to reduce the false positives and negatives, and provide a more accurate boundary of the segmentation.
- Employing a CBF in the encoder-decoder structure to boost the features of the MRI.
- Reducing the complexity of the architecture by essentially replacing a whole stage of convolutional layers and filters with a relatively much simpler CBF.
- Benchmarking and evaluating the model on various segmentation metrics, against other state-of-the-art models for MRI segmentation.

## Related work

Image segmentation has been fundamental for most of the latest undertakings involving important image understanding tasks in computer vision. The basic operation of an image segmentation task is the division of the image into several disjoint areas based on features such as chromatic difference, geometric shapes and spatial texture. In general, there are two types of segmentation such as instance and semantic segmentation.

### Traditional image segmentation

Conventional methods can no longer be compared with the momentous performance shown by neural network-based models. Threshold-based segmentation^9^, region-based segmentation^10^ and edge detection-based segmentation^11^ are some of the categories which fall under conventional methods. These methods use a combination of knowledge extracted from the image using digital image processing and mathematical computation to segment the images.

### Neural network and deep learning based image segmentation

Segmentation using deep learning methods is the latest advancement in the field of image segmentation^12^. One of the reasons for this transition is the astounding improvement in segmentation accuracy. A fully convolutional network was the first attempt at using deep learning for the semantic segmentation of images. After that, there has been some work involved in creating custom networks such as U-Net^13^, Mask R-CNN^14^, RefineNet^15^ and DeconvNet^16^. They have shown comparatively better performance and predominantly accomplish the task by presenting the concept of transfer learning, which helps in processing the fine edges.

### Recent methods

Since 2012, the MICCAI BraTS challenge has continuously advanced brain tumor segmentation research, providing a multi-MRI dataset with both clinical and synthetically augmented scans, as well as clinically verified ground truth masks. Each year, researchers and data scientists contribute state-of-the-art models, with most approaches leveraging the flexibility of CNNs—a key factor motivating the novelty of this work. Zikic et al.^17^ developed an algorithm based on 2D-CNN where multi-channel 2D patches of size 19 × 19 through all the MRI slices were extracted. In the work, the convolutional layers used filters of size 5 × 5 and 3 × 3, one after another in the 2 layered network. For achieving multi-class results, the author used softmax as the final activation layer.

U-Net^13^ has been the most explored architecture in the last 5 years, and it is evident that U-Net with task-oriented modifications has resulted in achieving state-of-the-art performance and accuracy in segmentation tasks. For voxel-level segmentation, variants of VNet^18^ have been showing prominent results. AFTer-Unet^19^ proposed by Xiangyi et al. utilises the capability of convolutional layers to extract detailed features and the transformer’s ability to improve long sequence modelling with a consideration towards training with few parameters and less GPU utilization. TransUNet^20^ proposed by Jieneng Chen et al. is another combination of transformer with U-Net Architecture, where the author highlights the advantage of transformers to serve as a strong encoder module for medical image segmentation. This, when introduced within a U-Net, enhances finer localised spatial information. Qin et al. proposed U^2^-Net^21^ for the purpose of salient object detection and segmentation, which consists of a nested two-stage U structure based on U-Net. This, combined with their newly introduced ReSidual U block (RSU) which enables the network to gather more global as well as local information from both shallow and deep layers, irrespective of the resolution. Chenggang Yan et al.^22^ discussed the importance of deep multi-view enhancement for the retrieval of images. In^23–27^, the authors enlightened the crucial role of denoising the 3D images, face recognition, multi-feature fusion and the importance of evaluating the quality of the images when the reference images are not available.

Recent advancements can also be seen in instance segmentation of brain tumor into sub-categories by colour coding, which has been achieved by Jindong Sun et al.^28^. This work also focuses on implementing custom neural network modules, which brings in novelty and results in improved efficiency. In Attention Res-UNet with Guided Decoder (ARU-GD)^29^, attention gates were introduced as primary component in UNet, which the authors claim to have reduced the irrelevant activation, this enables the model to focus on features relevant to the tumor segmentation task which in turn helps reducing the false positive and improve the final outcome.

Wang et al. proposed a two-stage medical image segmentation approach that includes image fusion as well as image segmentation^30^. Although not exactly a multi-modal fusion, the authors suggest that the T1C and FLAIR versions of the MRI from the BraTS dataset be fused, and the tumor be segmented from it further. The suggested Multi-Feature Grey Wolf Optimizer (MFGWO) technique allows Pulse Coupled Neural Networks (PCNN) to establish parameters automatically and achieve the best segmentation parameters during the image segmentation stage. Furthermore, by merging several hybrid image features, the fitness function of MFGWO increases the segmentation performance of PCNN. Nonetheless, the settings of PCNN are specified manually using prior information. It is, however, a challenging task to figure out how to automatically configure the model parameters to achieve the best segmentation performance. From the literature survey, some of the research gaps have been identified, and these gaps motivated us to implement the proposed technique. Section 2.4 outlines the current research gaps in deep learning techniques.

### Research gaps

- Despite their popularity, encoder-decoder-based architectures have some areas where research is still in progress, such as lowering the incidence of false positives and false negatives.
- Encoder–decoder-based models also occasionally had trouble identifying finer boundaries, resulting in segmentations that were crooked or erroneous.
- The requirement of long training time, excessive usage of memory and GPU are some of the main research gaps that have been observed in the recent methods that employ deep learning.
- Lack of contextual clarity is another limitation which has been a major constraint found in general deep learning models.

## Proposed work

In order to segment tumors from MRI images, a 4-staged 2D VNet ++ based research work is proposed in this work. Along with describing the specific loss function utilised, it also discusses the overall architecture for feature extraction and reconstruction that has been presented. This leads to the basic question: why use a CNN model? As discussed in the related work section, when compared to traditional models, CNNs can self-learn the hierarchical features such as edge information and textural details. This is a huge difference from the classical methods, which have manual feature extractions. CNN is robust to noise and variation and is scalable with the available data to improve the performance. When compared to transformers, which need large datasets and heavy augmentation, our focus on data-efficient, compact model design favours CNNs to reduce computational complexity. Additionally, replacing the 5th stage of the V-Net encoder-decoder architecture with a context boosting framework (CBF) streamlines processing and making the model well-suited for embedded and real-time medical applications and efficient for clinical portability.

The developed CNN frameworks are explained in depth in the following subsections, with an emphasis on the key features and benefits of those blocks and operations. The proposed model performs more accurate segmentation. This helps medical practitioners to understand the medical images more precisely and supports medical decision-making.

This research focuses on improving the quality of tumor segmentation by using a context-boosting framework to enhance the texture and deep features in the image. This, paired with a custom loss function tuned to learn the hard examples during the training of the model, generates the state-of-the-art results. In short, the focus of this paper is:

- Utilizing a specialized framework, CBF for boosting the contextual information in the MRI images.
- Reducing the complexity of the overall model by eliminating a whole stage of convolution layers and replacing them with the CBF.
- Using a customized LCFT loss function for guiding the training and for segmenting the tumors precisely.
- To support medical decision-making by helping medical practitioners study the MRI images more methodically.

### Context boosting framework (CBF)

**Fig. 1:**
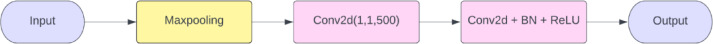
Context boosting framework (CBF).

**Fig. 2:**
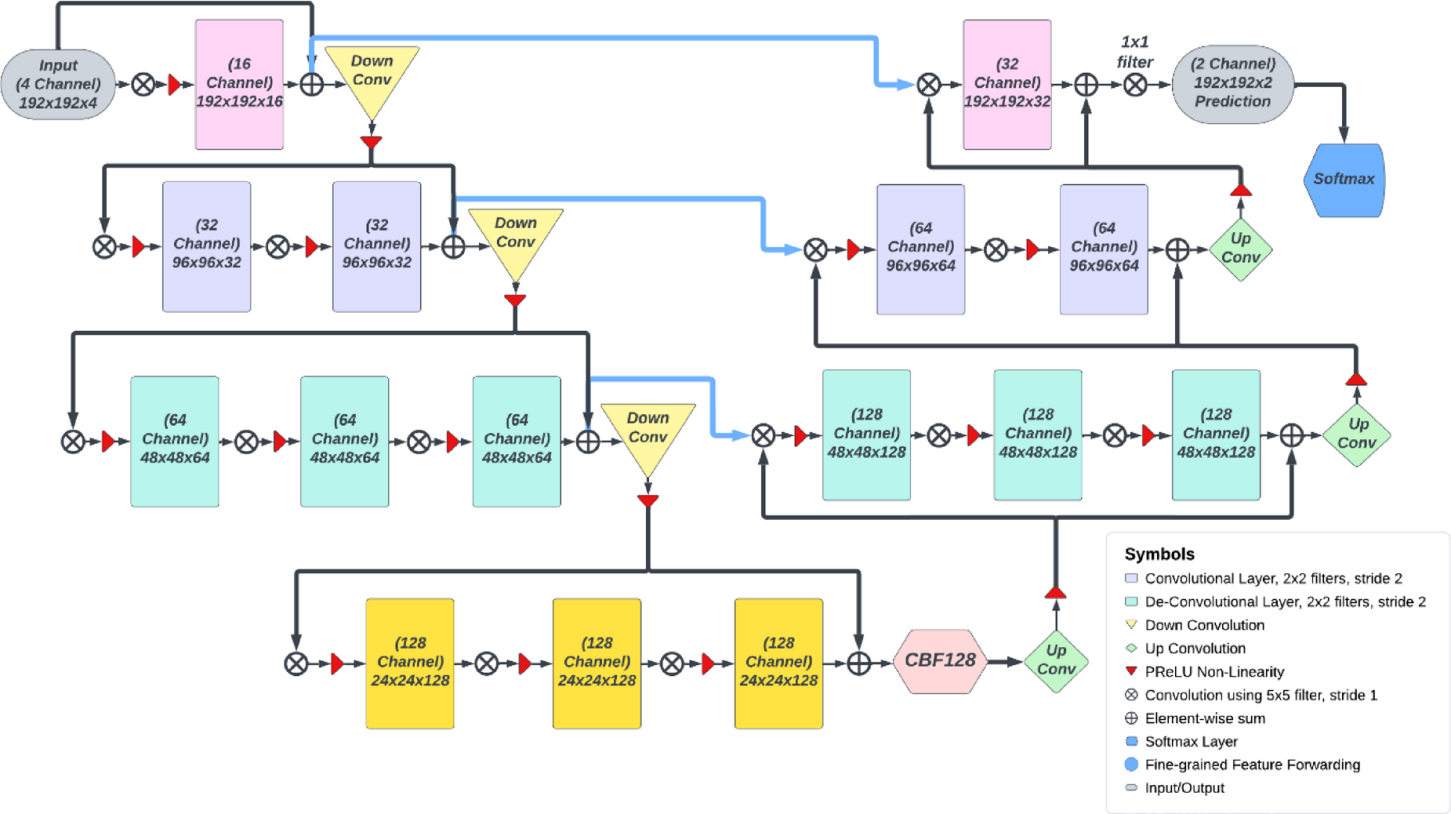
Architecture of proposed 4-stage VNet++ with CBF.

Context boosting framework is implemented at the deepest level of the CNN since contextual information is extracted only in the deeper layers. In this module, pixel-wise enhancement is done, mainly in the 500-filter Convolutional layer as depicted in Fig. 1. The contextual data is extracted and enhanced, which will, in turn, reduce the functional loss of the network in all iterations. Hence, the proposed model can segment the tumors effectively, even if they don’t conform to typical morphological characteristics. The general block diagram of CBF is presented in Fig. 2. The number of filters in the Convolutional layer was fixed at 500 after repeated tests and analysis. Values above 500 enhanced the noise in the images and classified an increasing area of normal regions as tumorous, while values below 500 did not classify enough tumorous regions as tumorous and labelled them as normal. The MaxPooling operation is done so as to reduce the complexity and the load on the model since a Convolutional layer with a large number of filters follows it.

### 4 stage: 2D VNet++ network architecture

The proposed model for segmentation is based on the 2D-VNet architecture. 2D-VNet is a customized version of the original VNet, which was designed for the segmentation of 3D medical images directly. 2D-VNet is an encoder-decoder style architecture that is 5 stages deep, with each stage containing as many convolutional filters as the stage number. It uses a conventional Dice loss to guide the training process, and takes longer to accomplish it due to the increased number of convolutional filters.

The proposed segmentation architecture consists of a 4-stage V-shaped structure with a CBF at the end of its deepest stage. It can be roughly divided into the downsampling side and the upsampling side. The regularization parameters used in the model were dropouts with a keep probability of 0.1, a learning rate of 0.0002, along with a Nesterov-accelerated Adaptive Moment Estimation optimizer and the LCFT loss function. The proposed architecture is shown in Fig. 2.

On the downsampling side, the 4 available varieties of MRI images (T1, T2, T1 CE and Flair) are appended to get a 4-channel data block that is passed to the network as the input. Prior to this, the images are resized or cropped from 240 × 240 pixels to 192 × 192 pixels. This is done in order to reduce the background noise and simply discard unnecessary information outside the region of interest (ROI) so that the CNN can work more efficiently and not perform unwanted computations. The first stage has a single convolution operation, the second stage has two and the third and fourth stages have three convolution operations. At the end of the convolution operations, an element-wise sum is taken with the initial input, and this is passed onto the subsequent downsampling block. This is done so that the information extracted at each stage makes it to the succeeding stage and is not lost, as the context-boosting framework requires maximum information. This sum is also fed forward to the corresponding stage in the upsampling side so that the upsampling side can use it during reconstruction. At the end of the downsampling side, the model object will have a depth of 128 channels.

At the end of the deepest stage, a good amount of contextual information has been extracted. This is the point where the CBF is introduced to enhance this contextual information. The output from the CBF is given to the right side of the network. The right side of the network consists of 3 upsampling stages, with each stage having the same number of convolutional operations as its counterpart on the downsampling side. The output of each stage has a channel depth 2 times that of its corresponding downsampling side. This is because in each stage, it takes in residual information from the corresponding stage in the downsampling side and concatenates it with the input it gets from its predecessor block. The upsampling side works in a similar way as the downsampling side, using element-wise addition with the output of the previous stage. The only key difference is that the operation done is transpose convolution instead of normal convolution, so that the deconvolution takes place. At the end of the upsampling side, there is a softmax layer that classifies each pixel of the output image into 4 categories according to the category of tumor, as represented in Fig. 2. Alternatively, this can simply be a two-class classification of the pixel into tumorous and non-tumorous.

### Custom log Cosh focal Tversky loss function (LCFT)

A modified loss function was used in the backpropagation to train the above network instead of a simple Dice loss. The original 2D-VNet used Dice loss for training the model, and the results were not up to the mark. After lots of trial-and-error with several loss functions, LCFT was concluded as the optimum choice. The modified loss function is a weighted combination of 3 loss functions—the Log Cosh Dice loss^8^, the Focal Tversky loss^7^ and the Jaccard similarity loss. This is different from conventional loss functions since it takes a weighted combination of three different loss functions. It reduces the number of false positives and false negatives, and is reflected in the accuracy of the model in the ablation study.

The Log Cosh loss function is an adaptive loss function that can be used in the case of highly skewed datasets. The Focal Tversky loss is an integration of focal loss with Tversky loss^31^, which is in turn a variation of the Dice coefficient^32^. Focal Tversky loss helps learn the hard examples, like the ones with small regions of interest (ROI). For the purposes of this research work, the Log Cosh part of the total loss function ensures that the skewness of the dataset does not affect the training process, at the same time bringing all the benefits of using a simple Dice loss function. The Focal Tversky part of the total loss function ensures that small regions of tumor that are located away from the main mass are also learnt by the model. The Jaccard loss has half the weightage as the two other losses, and is mainly used because Jaccard similarity index or IoU is what the models are mainly trained and evaluated on. The loss function used can be defined as in Eq. (1).

1 $$\:{L}_{total}\:=\:\alpha\:*{L}_{LC-Dice}\:+\:\beta\:*{L}_{Focal\:Tversky}\:+\:\gamma\:*{L}_{Jaccard}$$

Here $\:{L}_{LC-Dice}$ is the Log Cosh Dice Loss, $\:{L}_{Focal\:Tversky}$ the Focal Tversky loss, and $\:{L}_{Jaccard}$ the Jaccard loss. Since the total loss should be a value less than 1, simply adding the 3 separate losses would give the total loss out of 3, which is not useful. After several experiments with varying weights, the variables α, β, and γ were fixed as 0.6, 0.2 and 0.2, respectively. The $\:{L}_{LC-Dice}$, called as the Log Cosh Dice Loss and is defined as given by Eq. (2). Dice loss $\:{L}_{Dice}$, which is the basis of the Log Cosh dice loss, is defined in Eq. (3).

2 $$\:{L}_{LC-Dice}\:=\:loglog\:\left\{coshcosh\:\left({L}_{Dice}\right)\:\right\}\:$$

3 $$\:{L}_{Dice}\:=\:1-\frac{2*intersection}{union+intersection}\:=\:1-\frac{2TP}{2TP+FN+FP}$$

Here, TP stands for true positive, FN stands for false negative, and FP stands for false positive.

$\:{L}_{Focal\:Tversky}$ in Eq. (1), refers to the Focal Tversky loss, which is a combination of the focal loss and Tversky loss based on Tversky index^31^, depicted by Eq. (4).

4 $$\:{L}_{Focal\:Tversky}={(1-Tversky\:Index)}^{\gamma\:}$$

Here γ is a hyper parameter typically fixed as 0.75, and the Tversky index is defined as given by Eq. (5).

5 $$\:Tversky\:Index=\:\frac{TP}{TP+\alpha\:FN+\beta\:FP}$$

Note that when α and β are both 0.5, the definition of Dice coefficient^32^ is obtained, and when both are 1, the definition of Jaccard index is obtained. Setting the value of α greater than that of β, the false negatives are penalised more. This simply means that the model is heavily penalised if it predicts a tumorous pixel as non-tumorous, since this can adversely affect the diagnosis and even the life of the patient. Finally, the Jaccard loss $\:{L}_{Jaccard}$ is defined in Eq. (6).

6 $$\:{L}_{Jaccard}=1-\frac{TP}{TP+FP+FN}$$

## Results and discussions

The aim of image segmentation in this article is to segment the tumorous regions from MRI images, which have their structural information enhanced, using the CBF module and the LCFT loss function. The proposed segmentation network 4-stage 2D VNet++ was trained and tested on the MICCAI BraTS2020 dataset^33^. This section summarises the several tests carried out using the input dataset to assess the performance of the deep learning network.

### Dataset and environmental configuration

The BraTs 2020 dataset, comprising 259 cases of high-grade glioma and 110 cases of low-grade glioma, is a multi-institutional pre-operative collection of MRI images of inherently heterogeneous brain tumors. The image sets are already de-noised and registered, meaning they have been brought to a single coordinate system such that they superimpose on top of each other perfectly. Each 3D image has a dimension of 240 × 240 × 155 voxels. Each case was divided into 4 categories, namely (a) native (T1), (b) post contrast T1-weighted (T1Gd), (c) T2-weighted (T2) and (d) T2 Fluid Attenuated Inversion Recovery (T2-FLAIR), which were acquired with different clinical protocols and various scanners from multiple data contributing institutions.

The 4-stage 2D-VNet++ architecture is trained on the BraTS 2020 dataset, out of which 20 patients’ 3-D images are used. The 20 cases were selected randomly since the computational and storage resources were limited to accommodate the entire dataset. This, in hindsight, may have affected the generalisability of the model, but does not affect the effect the novelty elements have. The dataset also contains the ground truth image for each MRI, which is manually marked by expert medical professionals. No data augmentation techniques were used during the training process. 80% of the images were used for training, and the rest 20% were used as the testing dataset. The dataset takes utmost care to follow clinical and ethical guidelines while acquiring the images, like maintaining the anonymity of the patient and making sure that no reverse engineering can be done to extract the same.

The model was developed using the Keras library, along with the help of the Tensorflow framework. All 4 varieties of MRI from the 20 patients were taken, and the vertical slices from 30 to 120 were extracted from each of them, making for a total of 1800 2D images for each of the 4 varieties of MRI. Before passing as input to the model, the four types of MRI were concatenated channel-wise to create a 4-channel data block, which has slices of resolution 192 × 192 pixels. The slices, which were originally 240 × 240 in dimension, were cropped to 192 × 192 by cutting off the edge pixels. These eliminated parts are not in the region of interest, thereby reducing the computational load on the model.

### Performance analysis

A performance comparison has been done with the recent work done by^34^ and other state-of-the-art techniques^29,35–40^ in image segmentation that employ deep learning models as portrayed in Table 1. A detailed ablation study of the proposed model is presented in Table 2, where the effect of introducing each component of the model can be seen in the final results. The segmentation network’s segmented output image, in contrast with the source MRI image and the ground truth, is illustrated in Table 3.

**Table 1:** Comparison of the proposed model with recent state-of-the-art models.

	Loss	Dice coefficient	Jaccard similarity	Tversky index	Accuracy
ARU-GD^29^	0.4069	0.9189	0.8499	0.7018	0.9979
2D-VNet^34^	0.0069	0.9192	0.9579	0.9930	0.9937
MultiResUNet^35^	0.2510	0.7490	0.6004	0.7423	0.9972
3D-UNet^36^	0.2586	0.7408	0.5900	0.7293	0.9964
2D UNET MODEL^39^	0.0732	0.9268	0.8803	0.9117	0.9664
LinkNet^37^	0.0062	0.9938	0.9213	0.9590	0.9942
3D-UNET^38^	0.4737	1.0	0.250	1	0.25
TransUnet^40^	0.5891	0.4515	0.2917	0.4515	0.4515
Proposed model (4-stage 2D VNet++)	0.0061	0.9928	0.9964	0.9974	0.9971

**Table 2:** Ablation study results of the proposed model.

Techniques	Loss	Dice coefficient	Jaccard similarity	Tversky index	Accuracy
2D VNET	0.00694	0.91926	0.95793	0.99305	0.99375
2D VNET with custom LCFT loss function	0.00655	0.98976	0.99485	0.99678	0.99646
2D VNET with CBF Module	0.00633	0.93393	0.96583	0.99366	0.99381
2D VNET—custom LCFT loss function—4 stages	0.00616	0.99051	0.99522	0.99693	0.99657
2D VNET—custom LCFT loss function—5 stages	0.4161	0.6386	0.4701	0.6386	0.913448
Proposed model (2D VNET—custom LCFT loss function—CBF module—4 stages)	0.00614	0.99287	0.99642	0.99743	0.99717

**Table 3:**
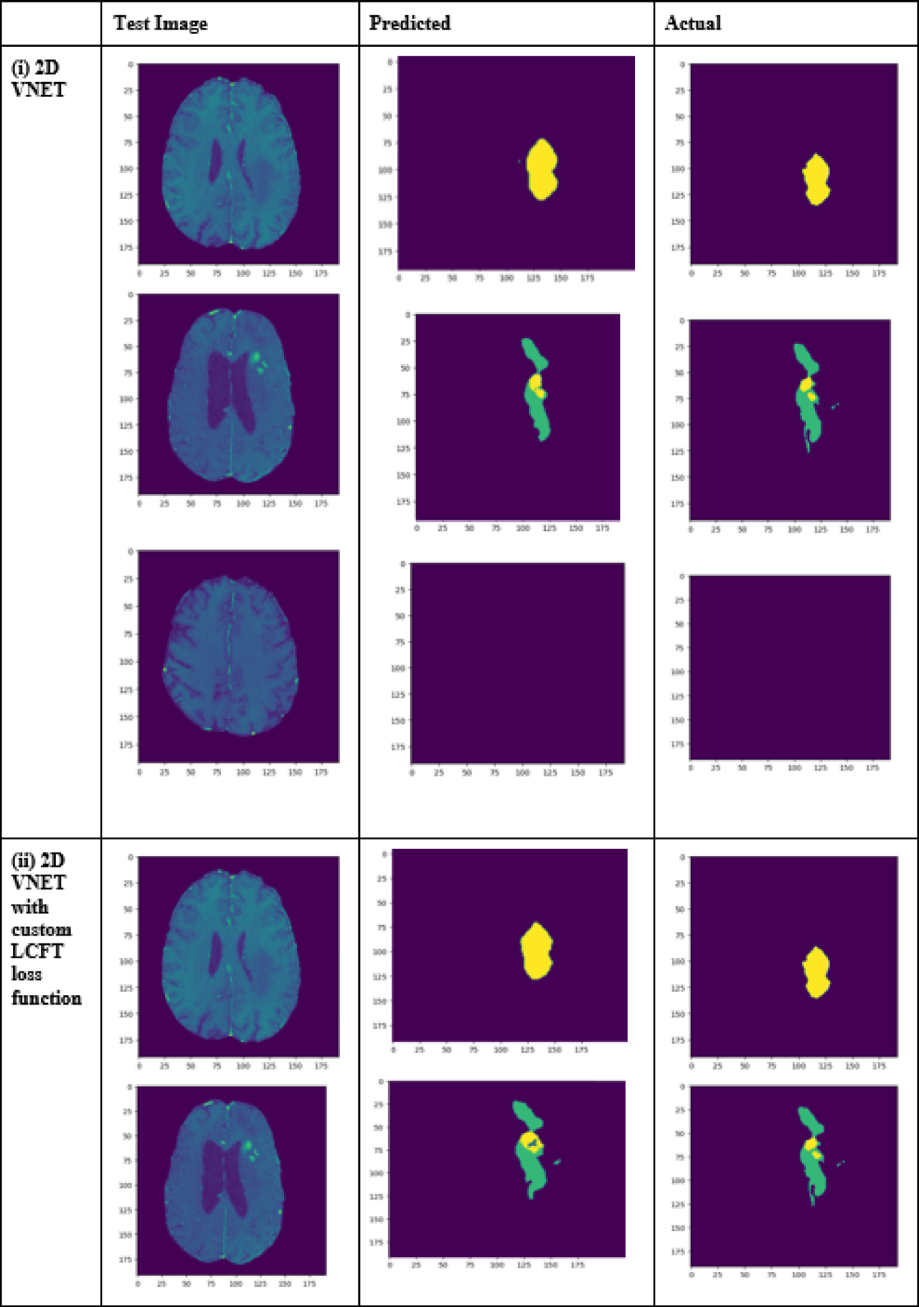

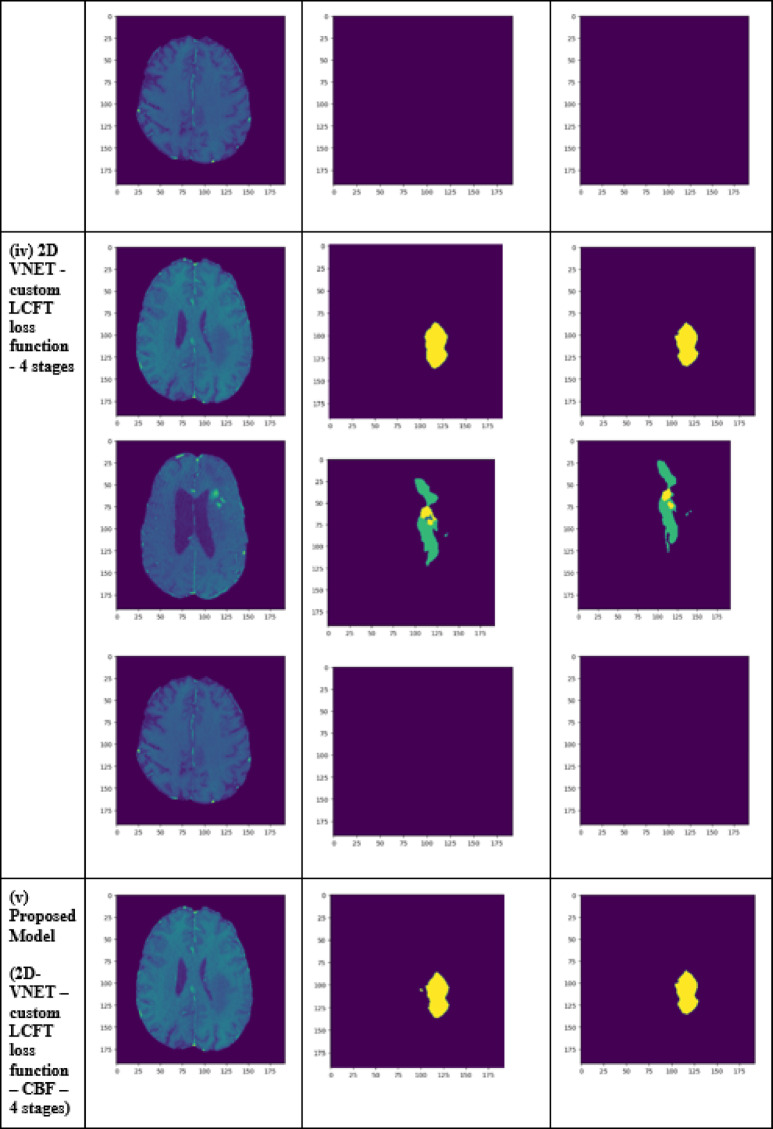

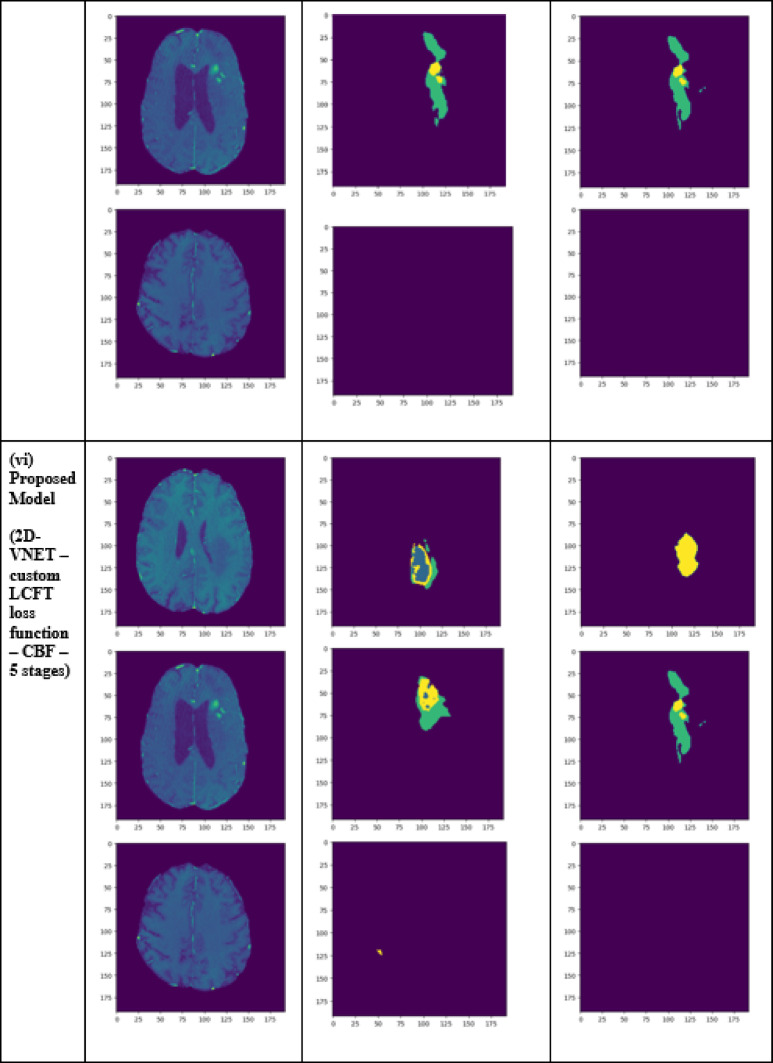
Visual comparison of the effect of introduction of a custom LCFT loss function – CBF – 4 stages in the proposed model.

In addition to the typical metrics testing accuracy and testing loss, the performance of the models was also evaluated on the basis of image segmentation specific metrics like Dice score^32^, Jaccard similarity index or IoU, as well as Tversky index^31^. The metrics Tversky index, Dice score and Jaccard similarity index are defined in Eqs. (5), (7) and (8), respectively. In machine learning and data science F1 score is the evaluation metric which monitors the presence of false positives (FP) and false negatives (FN) during the training process. Similarly, in Deep Learning Dice coefficient is used to monitor FN and FP during the training and evaluation.

The Dice score is a simple and useful summary measure of spatial overlap, which can be applied to studies of reproducibility and accuracy in image segmentation. It is defined as the harmonic mean of precision and recall. It also penalizes false positives, which is a common factor in highly class-imbalanced datasets like medical image segmentation. Hence, Dice score is one of the important metrics used for the evaluation of segmentation models.

Jaccard similarity index is computed using the number of true positives, false positives and negatives and the formula for the same is mentioned in Eq. (8). As it measures the amount of overlap between the ground truth and predicted segmentation, this performance metric is universally accepted for image segmentation process. In addition, the Tversky Index penalizes the false negatives more than the false positives. Since in tumor segmentation, missing a tumorous pixel can be considered more severe than falsely predicting a tumor. The Dice score is a universally accepted measure of spatial overlap, which is a metric for measuring the accuracy in image segmentation. All the models were trained for 30 epochs using the same dataset of 1800 images for each variety of MRI taken from the BraTS2020 dataset. This was fixed based on the timeout and RAM and GPU restrictions of Google Colab.

7 $$\:Dice\:Coefficient=\frac{2TP}{2TP+FN+FP}$$

8 $$\:Jaccard\:Similarity\:Index=\frac{TP}{TP+FP+FN}$$

From Table 1, notice that the proposed 4-stage 2D VNet++ performs better in terms of all three segmentation-specific metrics, namely, the Dice score, the Jaccard similarity index and the Tversky index. The improvement in the Tversky index indicates that the proposed model is better at avoiding false negatives, as it penalises itself more during the training process for the false negatives as compared to false positives. This enhances the model’s ability to detect maximum tumor area and not classify a tumorous pixel as non-tumorous, since this could lead to grave consequences in the medical field in practical application. Overall, the proposed model shows a slight decrease in the loss as compared to state-of-the-art techniques like 2D-VNet by 11.59%. Whereas ARU-GD shows an unusually high loss value, probably because the model should ideally be trained using a much larger dataset, which is typically difficult and impractical to obtain in the medical domain. The proposed model also performs better in terms of Dice score, Jaccard similarity index and Tversky index by an average of 8.02%, 10.23% and 17.7% respectively. Table 4 provides a visual representation of the predictions made by recent state-of-the-art models.

**Table 4:**
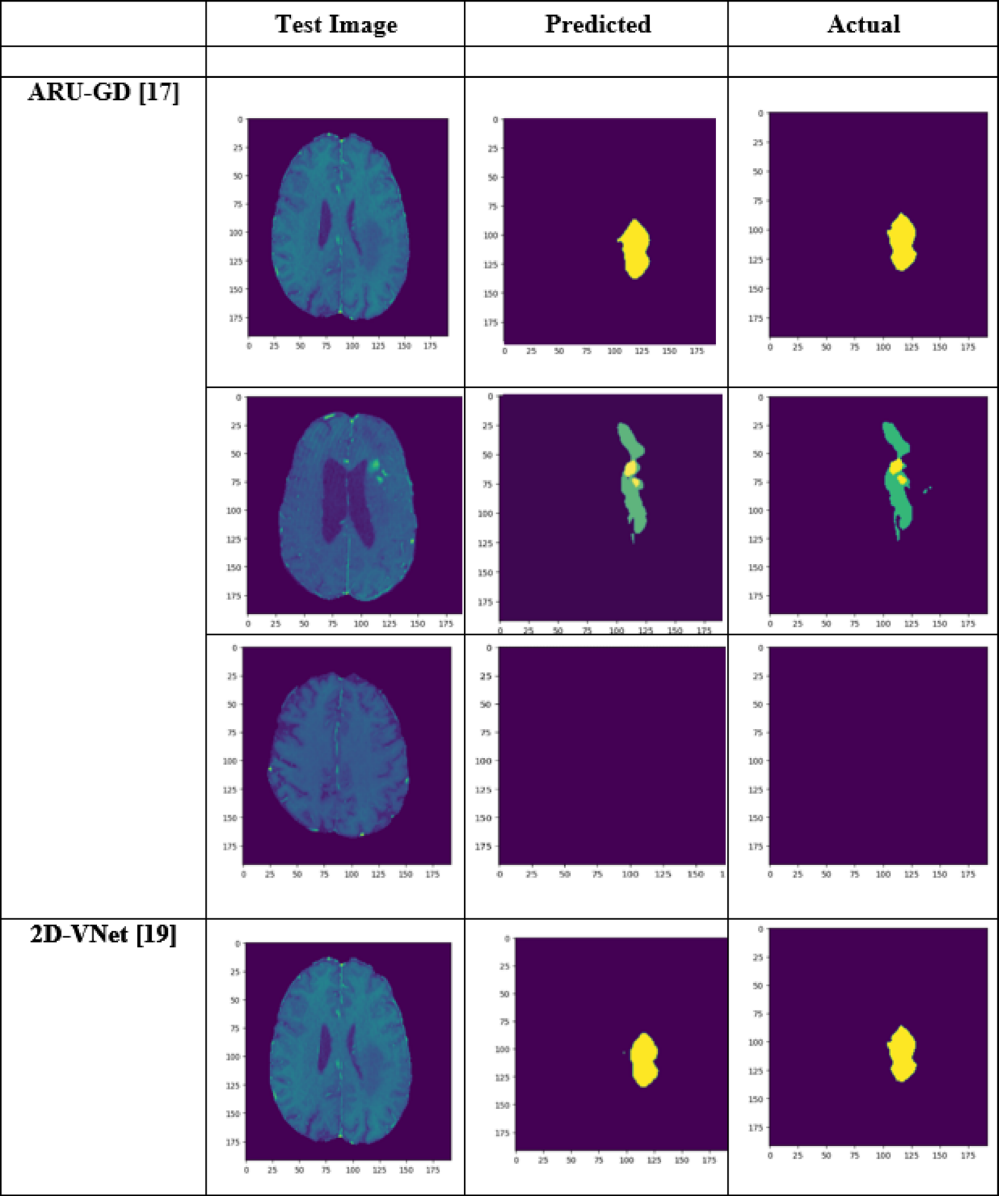

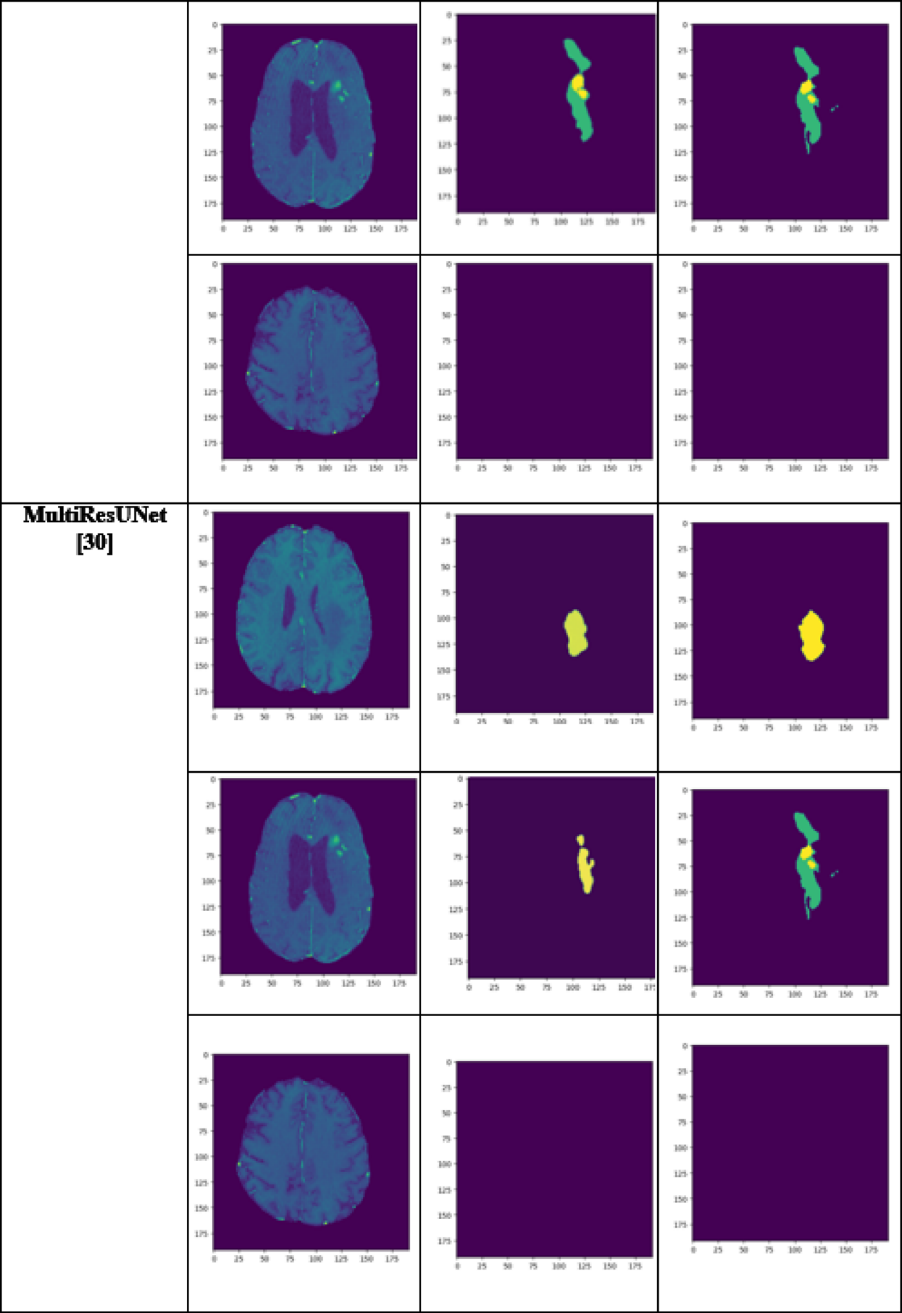

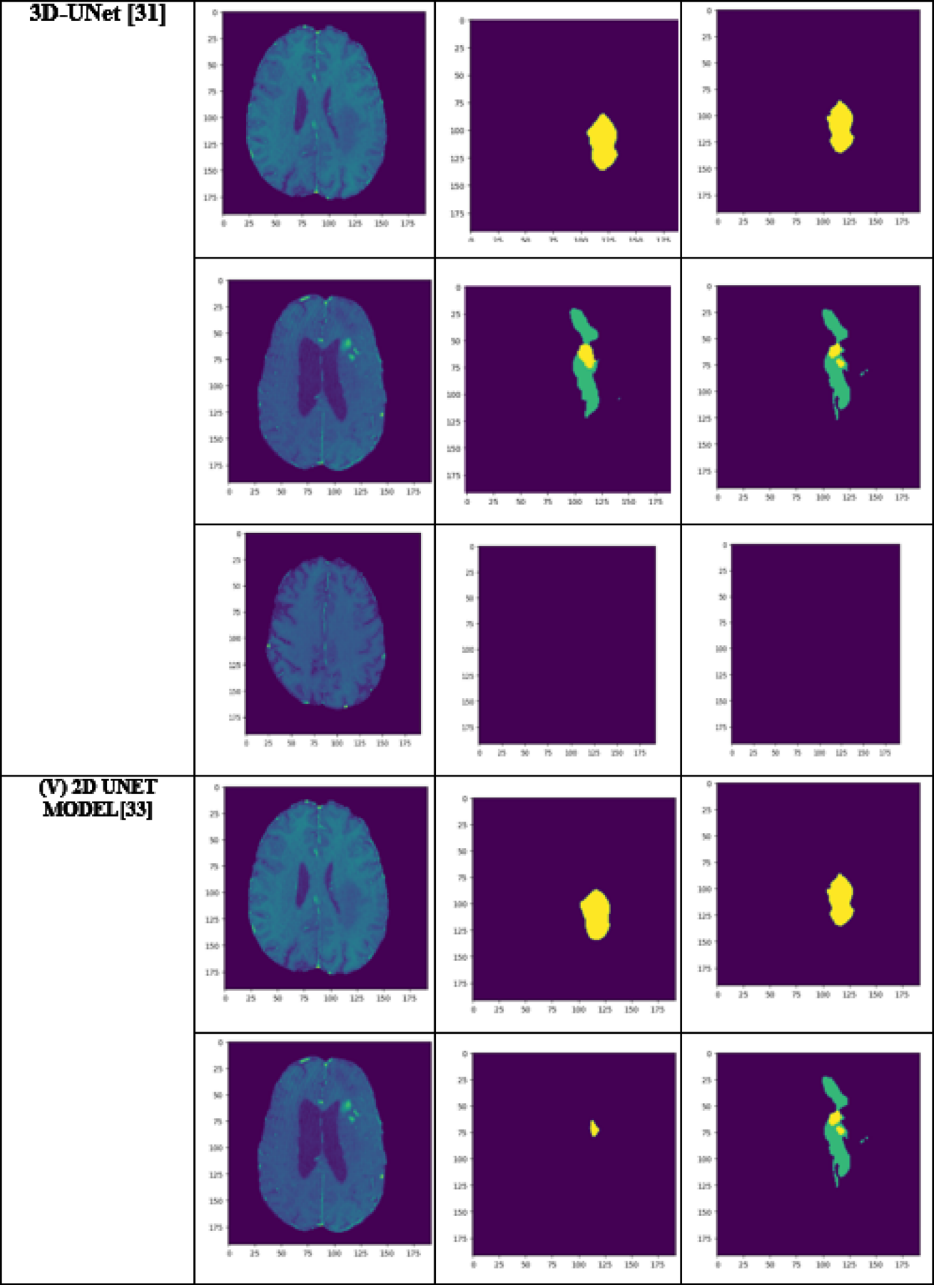

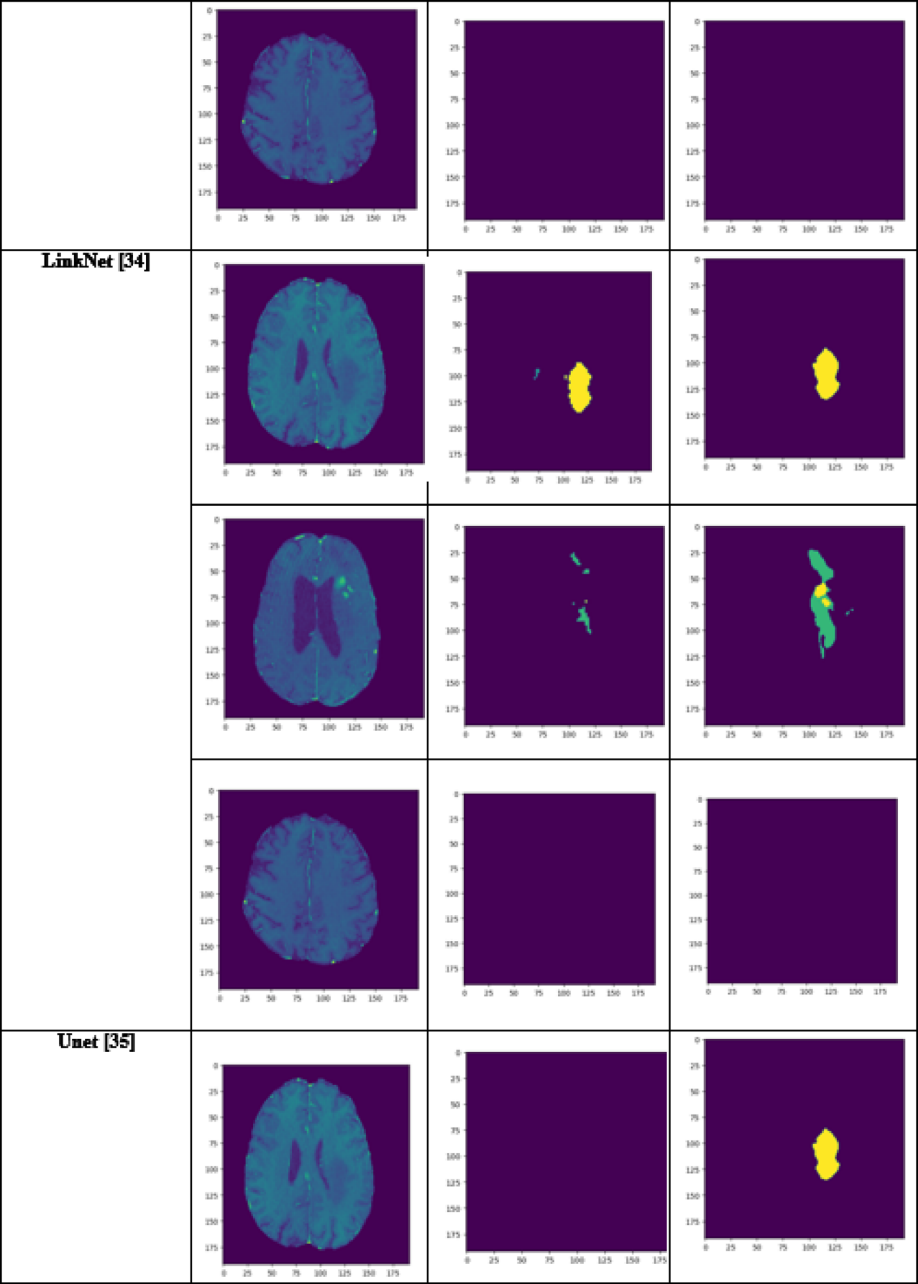

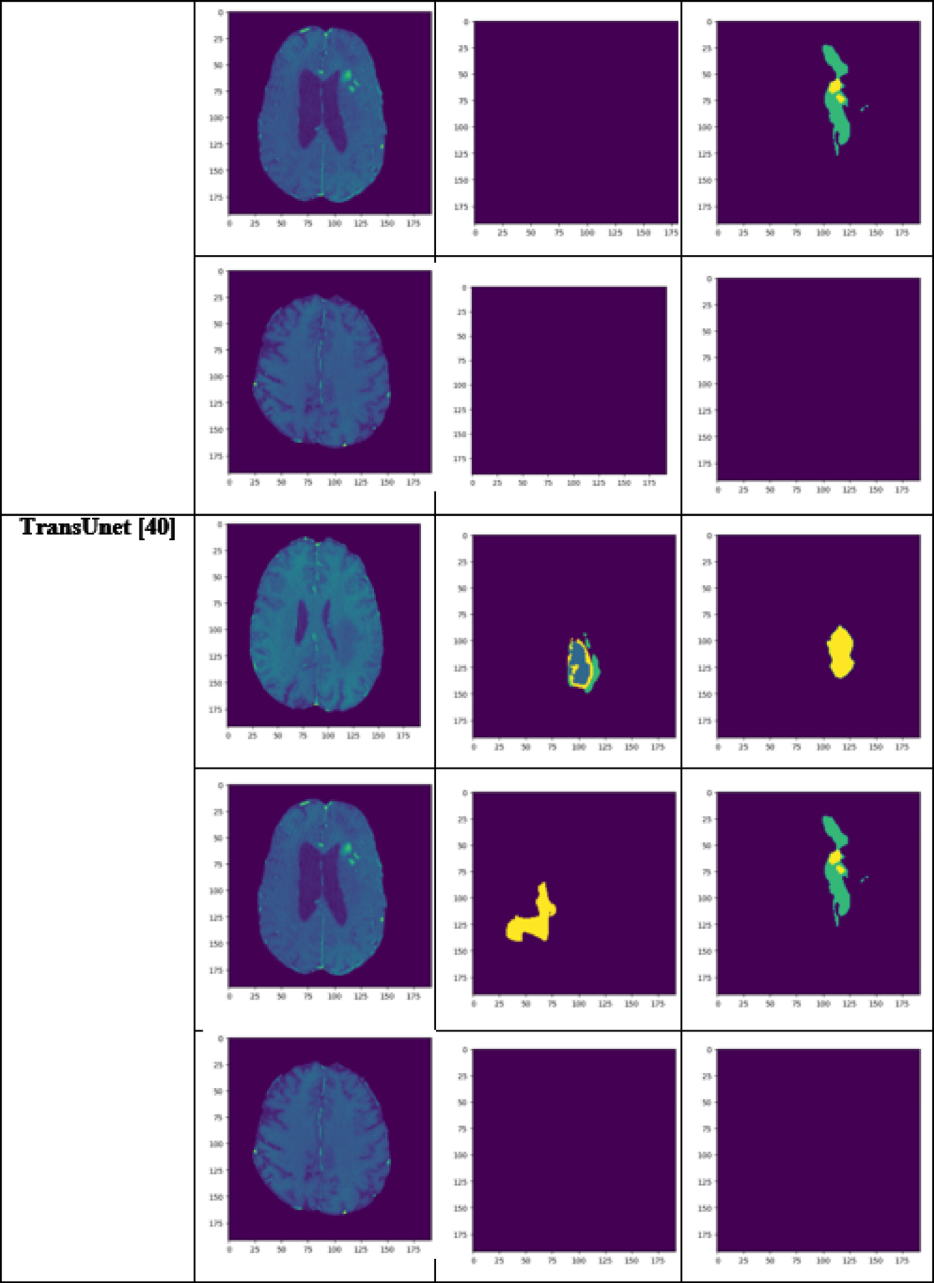

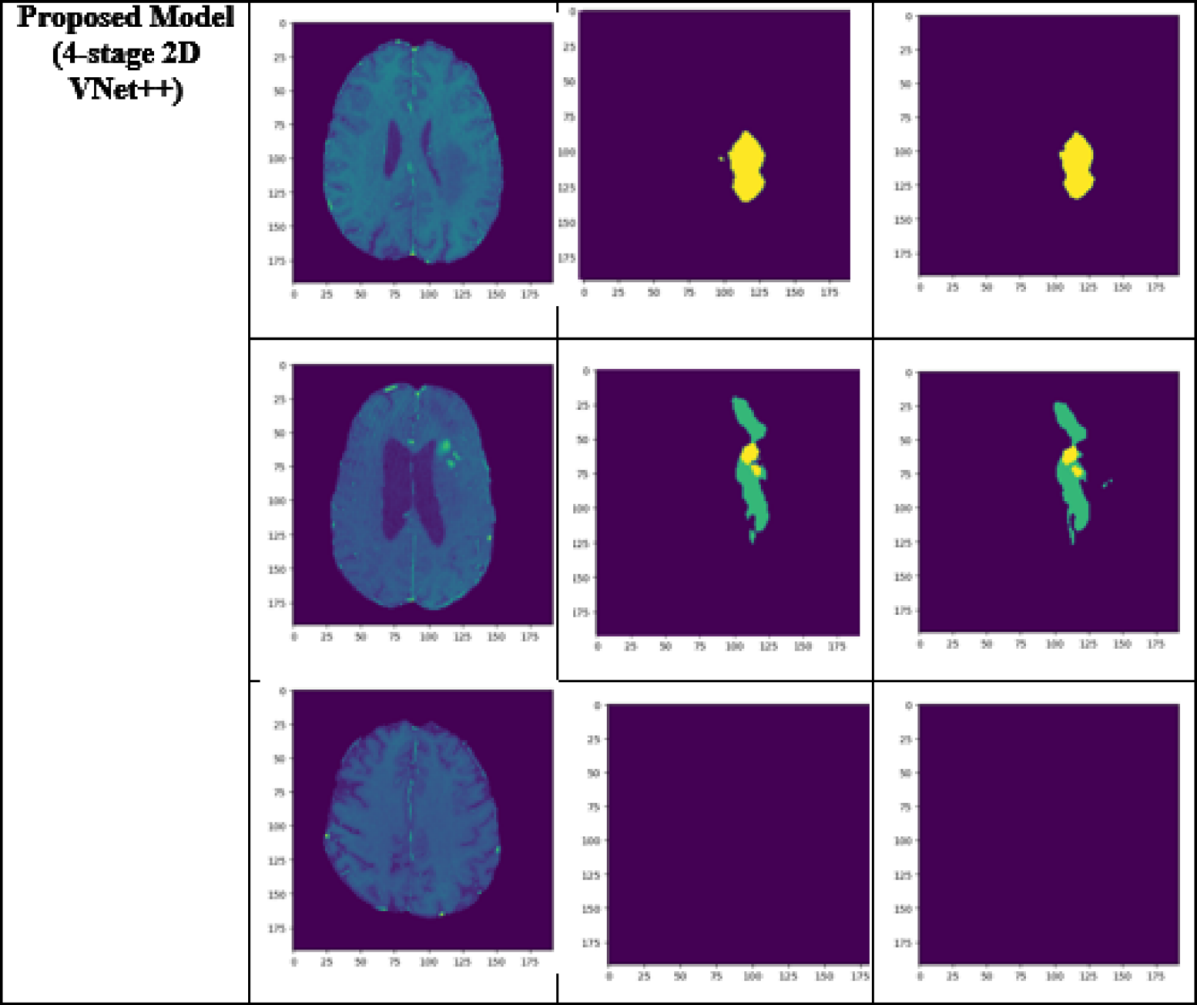
Visual comparison of predictions with recent state-of-the-art.

One-way ANOVA was performed to statistically support the excellent performance of the model. The null hypothesis can be given as—“There is no significant difference in the performance metrics of the proposed model compared to other state-of-the-art models”. The f-statistic was calculated to be 3.1398, and the p-value was computed to be 0.0461. Since p-value < 0.05, it can be concluded that there is a significant improvement in the performance metrics of the proposed model when compared to other state-of-the-art techniques.

To analyze the efficiency of the proposed model, it is also tested on BraTS2019 dataset. The visual comparison analysis is shown in Table 5. It illustrates the segmentation network’s segmented output image, in contrast with the source MRI image and the ground truth. Table 2 presents a detailed ablation study of each component of the proposed model. It can be seen from column (ii) that the introduction of the custom LCFT loss function replacing the typical Dice loss showed great improvement in all three segmentation-specific metrics. From Tables 3 and 5, it is observed that the custom LCFT loss function had the most significant impact on the performance of the proposed model. This is because it helps in reducing the overall false positives and false negatives in the segmented image.

**Table 5:**
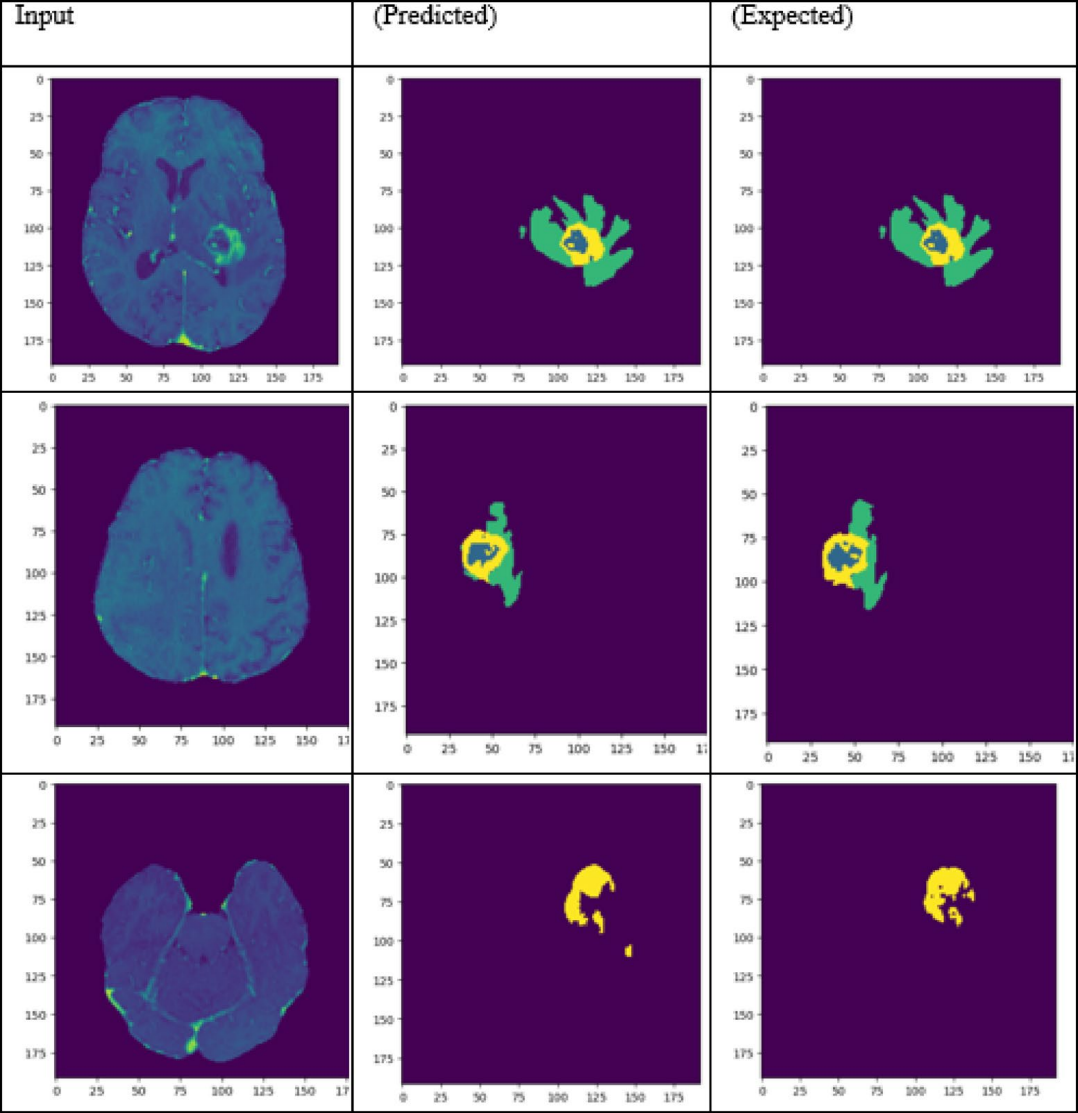
Visual comparison of predictions of the proposed model for BraTs2019 dataset.

The introduction of the CBF in the middle of the original 5-staged 2D-VNet can also be seen as bringing significant improvements to the performance metrics, as depicted in column (iii). The reduction of the number of stages of the network from 5 to 4 reduces the total loss of the model significantly, from 0.694 to 0.616%. Naturally, it could hence be mistakenly assumed that if the number of stages is reduced to 3 or 2, the model could reduce the loss even more. But as it turns out, since the contextual information is present only in the deeper layers, some level of depth is necessary so that the CBF can extract this contextual information. Hence, from the experimental analysis, it was concluded that 4 stages is the optimum depth. It is also to be noted that as a result of this reduction of stages, the training time of the model reduced from an average of 330 ms/step to around 170 ms/step in each epoch. Finally, the proposed model, consisting of the custom LCFT loss function, the CBF module and a reduced number of stages, is found to give the best results across all five metrics.

A few test images from each of the five conditions are shown in Table 3, for a side-by-side comparison of the predicted image of the model and the actual ground truth. Even though the proposed model achieves excellent results, it is a limitation that a high-end GPU with a significant amount of memory and computation power is essential to train the model. Another drawback is that the batch size had to be reduced in order to carry out the training process with limited RAM, which may have introduced a generalization gap. This had to be overcome by other methods, like introducing dropouts and other generalization techniques. The overall parameters required for the 5 stages are 280.52 MB whereas the 4 stages model requires only 73.61 MB. The total inference time for the five stage network is 6534. 740 s whereas for proposed four stage model takes only 4230.856 s to train the model completely.

## Conclusions

This research article presents a deep learning model for the segmentation of brain tumors from MRI images. The primary objective of this research is to introduce customized frameworks and components to the typical encoder-decoder architecture for segmentation, and evaluate the performance with state-of-the-art techniques that employ some form of deep learning. Extensive experiments were conducted on state-of-the-art models like 2D-VNet and ARU-GD, and this work is the first of its kind that introduces a custom CBF altering the depth of the deep models for optimizing the outcomes. The outcomes of this research article clearly depict that the novel modules introduced contribute greatly to the improvement in the performance metrics. The proposed model outperforms the architectures to give a Dice score of 99.287, Jaccard similarity index of 99.642 and a Tversky index of 99.743, which is subject to certain limitations, such as reliance on high-end GPUs, a relatively small training dataset, and reduced batch sizes due to hardware and memory constraints. For real-world clinical applicability, further validation on diverse, prospective datasets and integration with clinical workflows are required, along with robustness assessments to ensure safety in deployment. Clinical translation will require prospective validation with multi-institutional MRI data, blinded comparisons with radiologists’ segmentations, and pilot testing within radiology workflows. Future research will focus on adapting the architecture for multimodal MRI input and optimizing it for real-time deployment on edge hardware, with the long-term goal of integrating our approach into clinical trials and decision-support tools for neuro-oncology.

## Future work

The segmentation model proposed has the ability to learn and adapt based on the dataset used in training, testing and validation. Since the motivation of this research is to develop a segmentation model for MRI images, the experimental hyperparameter fine-tuning has been done accordingly. The proposed model can also be used for segmentation of different kinds of medical images, with necessary changes to the hyperparameters and suitable preprocessing. In future, the authors have planned to implement segmentation process for various types of medical images and to incorporate multi-modal image fusion as a preprocessing to segmentation. The authors have also planned to integrate the proposed algorithm with machine learning algorithms for enhanced diagnostic analysis^12^.

## Data Availability

The datasets analysed during the current study are available in the^41^ repository, [https://www.kaggle.com/datasets/awsaf49/brats20-dataset-training-validation].
